# How Frontoparietal Brain Regions Mediate Imitative and Complementary Actions: An fMRI Study

**DOI:** 10.1371/journal.pone.0026945

**Published:** 2011-10-28

**Authors:** Brenda Ocampo, Ada Kritikos, Ross Cunnington

**Affiliations:** 1 School of Psychology, The University of Queensland, St Lucia, Australia; 2 Queensland Brain Institute, The University of Queensland, St Lucia, Australia; University of Bologna, Italy

## Abstract

The ‘mirror neuron system’ (MNS), located within inferior parietal lobe (IPL) and inferior frontal gyrus (IFG), creates an internal motor representation of the actions we see and has been implicated in imitation. Recently, the MNS has been implicated in *non-identical* responses: when the actions we must execute do *not* match those that we observe. However, in such conflicting situations non action-specific cognitive control networks also located in frontoparietal regions may be involved. In the present functional magnetic resonance imaging (fMRI) study participants made both similar and dissimilar actions within two action contexts: imitative and complementary. We aimed to determine whether activity within IPL/IFG depends on (i) responding under an imitative versus complementary context (ii) responding with similar versus dissimilar responses, and (iii) observing hand actions versus symbolic arrow cue stimuli. Activity within rIPL/rIFG regions was largest during observation of *hand* actions compared with arrow cues. Specifically, rIPL/rIFG were recruited only during the imitative context, when participants responded with similar actions. When responding to symbolic arrow cues, rIPL/rIFG activity increased during dissimilar responses, reflecting increased demands placed on general cognitive control mechanisms. These results suggest a specific role of rIPL/rIFG during imitation of hand actions, and also a general role of frontoparietal areas in mediating dissimilar responses to both hand actions and symbolic stimuli. We discuss our findings in relation to recent work that has examined the role of frontoparietal brain structures in joint-actions and inter-actor cooperation. We conclude that the specific brain regions identified here to show increased activation during action observation conditions are likely to form part of a mechanism specifically involved in matching observed actions directly with internal motor plans. Conversely, observation of arrow cues recruited part of a wider cognitive control network involved in the rapid remapping of stimulus-response associations.

## Introduction

In humans, frontal-parietal brain regions responding to both observed and executed actions form what is known as the mirror neuron system (MNS) [1], [2], [3], [4]. There is controversy surrounding the exact function of the MNS in motor priming. According to one hypothesis, mirror neurons are responsible for directly matching observed motor plans with those internally activated. This is considered to underlie our ability to learn imitative behaviours, and also to understand ‘from the inside’ the actions of others [5], [6]. Some have suggested that the MNS responds to the *goal* of an action rather than its physical properties, however, [4], [6], with recent evidence showing increased frontoparietal activation during non-identical actions [7]. It is important to determine whether performing actions that are dissimilar to those we observe are mediated by brain regions responding specifically to hand actions, or by general cognitive control mechanisms responsible for selecting and preparing conflicting responses.

Newman-Norlund and colleagues had participants observe and perform reach-to-grasp actions towards a manipulandum, using either a power or precision grip [7]. Their results revealed increased activation within rIPL/rIFG when participants prepared dissimilar (different grip-type) compared with similar (same grip-type) responses to observed actions. The authors concluded that the MNS responds to broad action goals rather than physical information relating to grip-type. An outstanding issue of the study by Newman-Norlund and colleagues, however, is the role of response-set in the modulation of frontoparietal brain regions. Specifically, in their paradigm similar and dissimilar actions occurred in two action contexts, ‘imitative’ or ‘complementary’, which established a mode of response based on task-rules. In the imitative context, participants mimicked the perceived action, whereas in the complementary context, they performed a dissimilar response by using the opposite grip-type. Although the authors interpret comparisons of similar versus dissimilar actions, the role of the action *context* was not directly examined.

It has recently been suggested that frontoparietal brain regions responding to action observation may reflect the simple associations required to link any sensory stimulus with an appropriate motor plan [8], [9] It thus remains unclear whether altering task-demands may have modulated activity within frontoparietal brain regions rather than action observation/execution per se. Therefore, cognitive control mechanisms required to form simple associations between a sensory stimulus and its appropriate motor response may explain these results [8], [9]. This possibility is supported by studies showing that the ‘automaticity’ of action priming can be reconfigured in favor of dissimilar responses when participants learn new sensory-motor pairings [10]. Furthermore, it is well established that the selection and planning of incongruent responses requires cognitive control mechanisms to select the appropriate, conflicting response and inhibit other responses that are more closely associated with a given stimulus [11]. The neuroanatomical substrates for these functions are also located within the inferior frontal gyrus (IFG) and the posterior parietal cortex (including IPL) [12], [13]. At present, we do not know whether frontoparietal involvement during observation/execution of dissimilar actions reflects the goal-specificity of the MNS, or the increased need for cognitive control (such as response-inhibition) in these circumstances.

In the present experiment we adopted a design similar to that of Newman-Norlund and colleagues [7]. Participants observed video segments depicting an actor's arm making reach-to-grasp actions towards a wineglass using either a power or precision grip. Two blocked contexts, Imitative and Complementary, determined whether participants responded with an action that was similar or dissimilar to the one they perceived on the screen, respectively. However, a small percentage of colour-cue trials forced them to violate the contextual rule in favour of a pre-determined action. Thus, both similar *and* dissimilar actions were performed in Imitative and Complementary contexts. Responses to these colour-cue trials were the focus of our analyses, because they revealed the unique effect of context, or response-set, in modulating activity within frontoparietal regions when performing similar versus dissimilar actions.

Furthermore, we asked whether brain activity during dissimilar actions reflects processes that respond selectively to hand actions. To this end, we added a condition in which participants responded to arrow cues (upwards and downwards-pointing arrows). Arrow cues were chosen as stimuli because like hand actions which prime imitative responses [14], [15], they intrinsically prime congruent directional movements, yet they are completely symbolic in nature [16]. We reasoned that performing similar responses to hand-action stimuli is akin to performing an action towards a similar direction as an arrow cue, whereas performing dissimilar responses to hand-action stimuli is analogous to performing an action towards a direction that is dissimilar to that indicated by an arrow cue. Thus we could determine brain activity that was specific to the observation of hand actions, compared with that which occurs for both hand actions and symbolic arrow cues.

If frontoparietal regions responding selectively to action observation are indeed involved in mediating dissimilar responses [7], then activity within IPL/IFG areas should be stronger when participants observe action stimuli compared with arrow stimuli, and when they perform dissimilar compared with similar actions regardless of the action context. On the other hand, if dissimilar responses are mediated by frontopairetal networks responsible for the rapid re-mapping of responses to all manner of perceptual stimuli, then IPL/IFG activity should be equally strong when observing actions and arrows. Importantly, frontoparietal activation should increase during dissimilar actions in the imitative context, but similar actions in the complementary context, as these situations are characterized by the greatest amount of response-conflict.

## Results

### Behavioural Data

We observed participants via infrared video monitoring during the scans, and recorded errors to ensure correct task execution. Errors in the Actions and Arrows conditions were very rare, accounting for less than 2% of trials. Due to technical problems, behavioural data were obtained from the same participants outside the scanner subsequent to fMRI testing. Participants' instructions did not differ from those received within the scanner.

**Figure 1:**
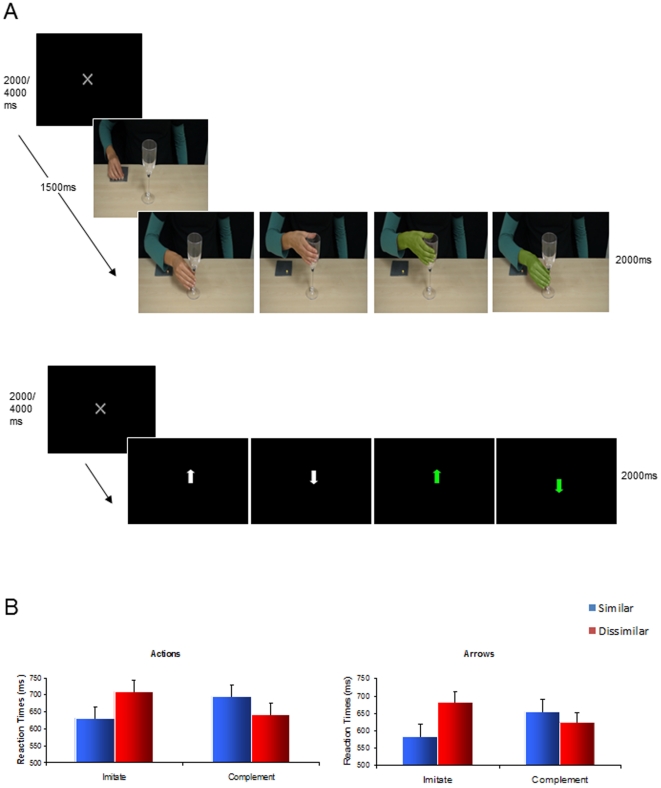
Trial sequences and behavioural results. A: Possible sequence of events in a given trial during the Actions (top) and Arrows (bottom) conditions. B: Reaction times (ms) for similar and dissimilar responses during Actions (left) and Arrows (right) conditions.

For both Hands and Arrows, there was a significant Context (Imitative/Complementary)×Similarity (Similar/Dissimilar) interaction (F(1,11) = 26.39, p<0.05; F(1,11) = 40.20, p<0.05, respectively). In the Imitative context, reaction times (RTs) were significantly faster when making similar compared with dissimilar actions across both Actions (M = 630.63, SD = 140.85; M = 709.45, SD = 69.42, respectively; t(11) = −4.60, p<0.05,) and Arrows (M = 582.56, SD = 80.85; M = 682.10, SD = 123.05, respectively; t(11) = −5.68, p<0.05) conditions. In the Complementary context, however, RTs were significantly faster for dissimilar compared with similar actions, for both Actions (M = 641.25, SD = 156.30, M = 695.83; SD = 144.30, respectively; t(11) = 4.93, p<0.05) and Arrows (M = 623.10, SD = 23.26; M = 653.98, SD = 83.18, respectively; t(11) = 2.84, p<0.05) conditions (see Fig. 1b).

To verify that neither action or arrow stimuli *per se* introduced specific biases in performance, we conducted a further 2 (actions/arrows)×2 (imitative/complementary)×2 (similar/dissimilar) repeated-measures ANOVA. This analysis revealed a significant main effect of Context (*F*(1,11) = 15.60, *p*<0.05) and a significant two-way interaction between Context and Similarity (*F*(1,11) = 62.80, *p*<0.05). Importantly, there was no main effect of stimulus, nor did this factor interact with either context or similarity.

### Neuroimaging Data

**Figure 2:**
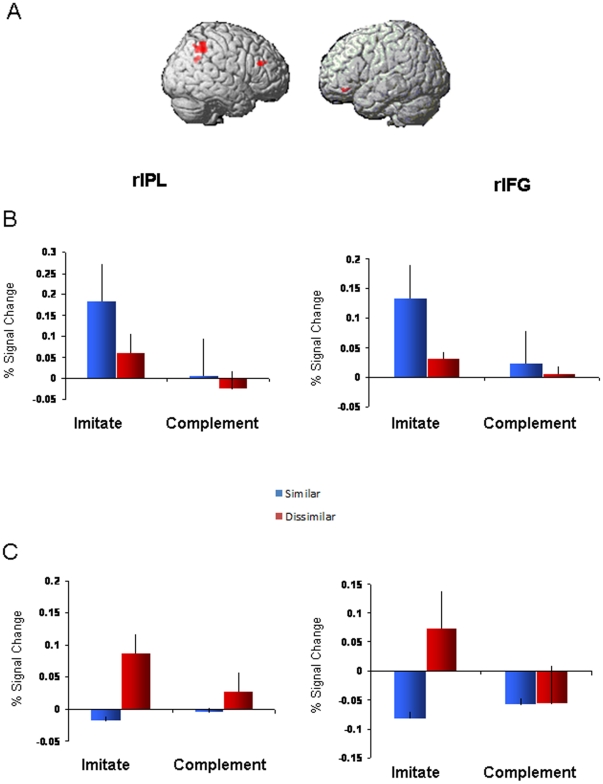
Imaging results. A: Significant interaction between Context and Stimulus located in rIPL (*p*<0.05, corrected) and second-largest cluster in rIFG (p = 0.174). B: % Signal Change for ROIs during Actions session. C: % Signal Change for ROIs during Arrows session. *Similar and dissimilar responses are compared with baseline (no-go) trials.

For the random effects group analysis, we entered the contrast of [dissimilar-similar] into a 2×2 factorial model with factors of Context (imitative/complementary) and Stimulus (actions/arrows). Crucially, there was a significant Context (imitative/complementary) by Stimulus (actions/arrows) interaction located within the right IPL (46, −48, 44, *p*<0.05 cluster-corrected, peak *Z* = 4.77; BA40). The second-largest cluster was in the right IFG (52, 32, 24, *p* = 0.174 cluster-level corrected; 58 voxels, with peak Z = 3.12, *p*<0.00005 uncorrected; BA46). As this cluster corresponds with the inferior frontal regions identified in previous studies [4], [7], [17], [18], [19] to be involved in perception-action linkages, and was the second-largest cluster identified here, it was also selected as a region of interest along with rIPL for further analysis (see Fig. 2a). The random-effects group analysis also revealed a significant main effect of Context; two clusters located within left superior frontal gyrus (−12, 40, 38, p<0.05 cluster-corrected, peak *Z* = 3.94) and right middle frontal gyrus (34, 50, 18, p<0.05 cluster-corrected, peak *Z* = 4.00) showed significantly greater activity in the Imitative compared with the Complementary context across both Actions and Arrows conditions. Given that our primary interest in the present experiment was to explore how observation of actions versus arrows modulated frontoparietal activation, however, we chose to focus our analyses on the Context×Stimulus interaction effect.

Levels of activation (% signal change) in the right IPL and right IFG clusters across Hands and Arrows conditions are shown in Figures 2b and 2c, respectively. Pair-wise comparisons of these % signal change values (using paired t-tests) demonstrate that during observation of actions, both rIPL and rIFG showed significantly greater activity when performing similar compared with dissimilar responses in the Imitative context (t(11) = 4.51, p<0.05 and t(11) = 2.27, p<0.05, respectively). In the Complementary context, however, activity did not differ significantly from baseline, nor were any differences found between similar and dissimilar responses (p>0.05). When participants observed arrows, activity within the rIPL and rIFG was significantly greater when performing dissimilar compared with similar responses in the Imitative context (t(11) = 2.20, p<0.05 and t(11) = 5.02, p<0.05, respectively). In the Complementary context there was again no difference in brain activity compared with baseline, and no differences between similar and dissimilar actions (p>0.05).

## Discussion

Consistent with previous studies [7], [10], [19], [20], we found response compatibility effects in RTs. Importantly, participants responded faster when making similar compared with dissimilar actions in the Imitative context, but also when making dissimilar compared with similar actions in the Complementary context. We obtained comparable results for the arrows condition: RTs were faster when participants performed similar actions (i.e., they followed the direction of the arrows) in the Imitative context, and when they performed dissimilar actions (reached in the opposite direction of the arrows) in the Complementary context. This suggests that the *prepotent* response, whether similar or dissimilar with the observed stimulus, is ultimately favoured. Although the pattern of results is consistent across Actions and Arrows conditions in the behavioural data, imaging results were markedly different between the two conditions, as discussed below.

In the Actions condition, rIPL and rIFG activity was significantly greater when making similar compared with dissimilar responses in the Imitative context. This indicates a direct motor mapping between observed actions and those represented in the participants' own motor system. Conversely, in the Arrows condition rIPL and rIFG showed the greatest level of activation when participants made *dissimilar* responses. In this condition, participants experienced the greatest degree of response conflict; they had to inhibit the ‘automatic’ response to follow the direction of the arrow cue and perform an action in the opposite direction. This is in stark contrast with the deactivation of rIPL and rIFG seen during *similar* responses to arrow cues. In such instances, inhibition of participants' automated responses was not required, and correspondingly, nor were extra efforts on behalf of cognitive control mechanisms.

It should be noted that the present study did not specifically map the mirror neuron system; this would require a localizer task that specifically defines brain voxels responding to both action performance as well as action observation (see, for example, Thioux et al., 2008). Furthermore, IFG results should be interpreted with caution given that this ROI was chosen based on its well-established involvement in perception-action linkages [4], [7], [17], [18], [19]. Nevertheless, our findings suggest that two separate processes might be involved in mediating perception-action linkages, both supported (either causally or correlationally) by frontoparietal brain regions. The first process, which *may* correspond with the mirror neuron system, operates within contexts that require similar responses, and serves to link perceived actions directly onto our motor system. Because it is specific to the observation of actions, this frontoparietal activity might allow an observer to represent others' actions at a simple motor level, enhancing access into higher-order interpretative processes. The second process allows an observer to voluntarily inhibit a response that is compatible with a stimulus, and prepare one that is compatible with task demands. As such, this ‘control’ network mediates conflicting responses during instances of increasing task complexity.

One possible limitation to this interpretation is that in the Complementary context, where participants' task-set was to perform dissimilar actions (opposite grip type or direction), the onset of a colour-cue trial eliciting a similar response did not significantly increase percent signal change in frontoparietal areas relative to dissimilar responses. This is also inconsistent with our behavioural findings indicating increased RTs during performance of similar actions in the Complementary context. A likely reason for this is that in the Complementary context, participants had adopted a response mode which was characterised by a constant inhibition of pre-potent actions. Therefore, a colour-cue eliciting a compatible action, although slowing responses relative to incompatible actions, did not necessitate any *extra* effort on behalf of response selection processes.

A distinction should also be drawn between the present paradigm, which provides a simple instruction to guide responses *without* extensive motor practice, and paradigms which purposefully train participants in stimulus-response associations [10]. It is possible that to recruit maximally the frontoparietal network for response selection/inhibition, learning must be far more explicit, with fewer variations in stimulus-response pairings than those present in this experiment. Future investigations are needed to explore this subject. It is also possible that in the current study there was no pre-potent, automatic response in the first place, and hence no change in brain activity. That is, in our task, frontoparietal regions may have been significantly activate *only* when participants made identical responses towards the static hands in the Imitative context. It remains the goal of future studies to determine whether cognitive control mechanisms operate equally across stimulus types for incompatible responses. Future studies should also consider opting for a greater sample size. The present study used 12 participants, and may have lacked the statistical power necessary to reveal differences between experimental conditions by means of whole-brain, voxel-wise contrasts.

Newman-Norlund and colleagues [7] reported increased mirror system activity for dissimilar actions. The novel aspect of this study is that we examined frontoparietal activation *separately* as participants made similar and dissimilar actions during Complementary versus Imitative contexts. We are therefore the first to show that response-set plays a crucial role in determining the extent to which action-selective mechanisms are recruited. Our study builds on previous work by suggesting that frontoparietal activation that is specific to action observation responds maximally when observing/executing similar actions, whereas dissimilar actions require the involvement of cognitive control networks that generalise across different types of visual stimuli. Nevertheless, it should be noted that the exact coordinates pertaining to IFG activation in the current experiment appear to be more anterior and superior compared with those identified by Newman-Norlund et al [7]. Therefore, differences in activation between the two studies may reflect the recruitment of distinct neural populations within similar brain regions. The sectors of IPL/IFG showing an interaction effect in the present study are not necessarily the same regions as those underlying dissimilar actions in Newman-Norlund et al.'s experiment. This may also be due to the different approaches to analyses employed by the two studies. Newman-Norlund et al. conducted only a conjunction analysis, combining effects of [different-same] across both Complementary and Imitative contexts to determine brain regions displaying a stronger BOLD signal during the preparation of dissimilar actions in both contexts. Our analysis, however, focused on differences between contexts and cue conditions. We specifically aimed to find areas in which the activation for “dissimilar” actions [the Dissimilar – Similar contrast] differed in Complementary versus Imitative contexts and for Action versus Arrow cue conditions, rather than conjoint or common activity across conditions. Our regions of interest were those brain regions that demonstrated an interaction effect between the action context (Imitative/Complementary) and the stimulus-type (Actions/Arrows) in a 2-way factorial model (see Materials and Methods section below). The results of the two studies must therefore be considered within the context of the two distinct methods of analysis.

It is interesting to note that recent work has implicated a functional dissociation between IPL and IFG regions in perception for action [21]. Activity within frontoparietal regions was measured using fMRI as participants played either a co-operative or a non-cooperative game in the scanner. Results showed that putative mirror neuron system (pMNS) regions involved during action observation and execution included premotor, parietal, and high-level visual areas. Importantly, however, pMNS regions did not display any *additional* activity during the co-operation task. Crucially, brain regions that showed a superadditive effect during the co-ordination of joint actions, which included areas within the IFG, were adjacent to (but not strictly part of) the pMNS. The authors suggest that the rapid re-mapping of visuo-motor associations during varying task demands recruits brain regions separate from the pMNS. This is consistent with a range of studies showing that regions located at the most rostral portion of the IFG are involved during the selection of motor acts that are appropriate to the task at hand; a mechanism that is crucial to many forms of sensory-motor behaviours [22], [23], [24], [25]. Although the present experiment did not seek to determine the dissociation between IPL and IFG during imitative versus complementary contexts, it is possible that the brain regions identified in the present study which responded primarily to action imitation are part of the pMNS recruited to translate between motor and visual codes, most evident in IPL areas. Frontoparietal activity identified by Newman-Norlund et al. during complementary responses, on the other hand, may reflect the flexible re-mapping of associations between perception and action-execution during interactive behaviours, a task likely to be carried out by IFG regions [21].

In conclusion, we show that observing goal-directed hand actions preferentially recruits rIPL and rIFG regions when performing similar rather than dissimilar actions, in a context where prepotent responses do not need to be inhibited. Conversely, when responding to symbolic arrow cues frontoparietal regions are preferentially active when performing dissimilar actions. This increase in activation may reflect the additional involvement of cognitive control mechanisms responsible for mediating responses to conflicting stimuli. We suggest that frontoparietal areas previously implicated in matching observed and executed actions are selectively engaged when imitating observed hand actions, whereas general cognitive control and response selection mechanisms mediate incompatible responses to all kinds of stimuli.

## Materials and Methods

### Participants

Twelve neurologically healthy volunteers participated in the experiment (mean age = 26.5, standard deviation = 5.7). All participants were right-handed, and had normal or corrected-to-normal vision. The study was approved by the Medical Research Ethics.

Committee of The University of Queensland. Written informed consent was obtained from all participants in this study.

### Stimuli

Whilst in the fMRI scanner, participants made reach-to-grasp actions towards a clear plastic wine glass (20 cm tall; 7 cm wide at the rim/1 cm at the stem) which sat vertically by their waist. The glass was attached to a plastic framework resting on the scanner bed. Participants continuously depressed a response button located where their hand naturally rested by their side, and reaction times were recorded as they released the switch in order to make their response. In two separate scanning sessions, they observed 2-frame picture sequences of either (i) a model's arm reaching to and grasping a wineglass, or (ii) centrally-presented upwards and downwards pointing arrows. Trials began with an inter-trial interval jittered at either 2000 or 4000 ms. The first frame of the action sequences consisted of a resting right hand positioned beside a clear plastic wineglass (1500 ms), and was followed by a second frame depicting the hand grasping the wineglass at the stem using a precision grip, or at the rim using a power grip (2000 ms). During colour-cue trials, the model's hand was shaded green on the second frame (see Figure 1A). Arrow cue trials were white arrows (1 cm wide×2 cm long) on a black background (2000 ms); during colour-cue trials the arrow was green. Stimuli were back-projected onto a screen situated at the foot of the scanner bed, and viewed by participants via a mirror mounted on the head coil. All stimuli were delivered using Presentation software version 12.0 (Neurobehavioral Systems, Davis, CA) run on a Dell laptop.

### Procedure

Within both Actions and Arrows sessions, participants responded according to the two blocked action contexts, Imitative and Complementary. In the Imitative context participants responded to observed actions by grasping the wineglass using a similar grip-type. For example, when observing the actor perform a precision-grip at the stem of the wineglass, they responded by similarly performing a precision-grip at the stem of the wineglass located by their side. In the Complementary context, they performed a dissimilar response (i.e., if they observed a precision-grip at the stem of the wineglass, they performed a power-grip at the rim). When responding to arrow cues they followed the direction of the arrow during the Imitative context by performing a power grip at the rim when the arrow pointed up and a precision grip at the stem when it pointed down. In the Complementary context they always reached in the opposite direction of the arrow by performing a power grip at the rim when the arrow pointed down and a precision grip at the stem when it pointed up. Therefore, responses were ‘similar’ when participants reached in the same direction as the arrow (up towards the rim for upwards arrows) and dissimilar when they reached in the opposite direction (down to the stem for upwards arrows).

Prior to each experimental block participants were instructed regarding their mandatory response to colour-cue trials, which occurred 40% of the time. During these trials participants were required to ignore the contextual rule (Imitative/Complementary) and perform a predefined grip (power *or* precision grip), specified before the commencement of the block. Incidentally, half of the colour-cue trials elicited a similar response and the other half elicited a dissimilar response. No-go trials were also included in order to prevent an expectation of movement. During the Actions condition, the model's hand went from a stationary position to a palm-lift, signifying an action ‘stop’; during the Arrows condition, the arrow was missing the arrowhead.

In the Actions and Arrows sessions, a total of eight experimental blocks/fMRI runs were completed. These comprised four Imitative and four Complementary contexts. The order of Context (Imitative/Complementary) and colour-cue meaning (power/precision grip) was counterbalanced across participants. There were a total of 80 trials per block; half of these were normal trials, where participants adhered to the action context; 40% were colour-cue trials, and the remaining ten percent were no-go trials. Twenty-five practice trials were completed before each scanning block. To prevent online response-switches, participants were asked to initiate their movement only when they were sure of which action they had to perform. Note that for our purposes, only the colour-cue trials were of interest. However, all conditions were modeled in the analysis.

### Behavioural Analyses

We analysed RTs to colour-cue trials, because these trials elicited similar *and* dissimilar responses in both Imitative and Complementary contexts. We conducted two separate 2×2 repeated measures ANOVAs for Actions and Arrows conditions, comparing factors of Context (imitative/complementary) and Similarity (similar/dissimilar). Planned comparisons were made between RTs for similar versus dissimilar actions in the Imitative context, and similar versus dissimilar actions in the Complementary context.

### fMRI Methods

Data were acquired on a 4-Tesla Bruker-Siemens MRI scanner at the Wesley Hospital, Brisbane. Head movement was minimized by using foam padding and never exceeded 3 mm in a run. For functional imaging, T2*-weighted echo-planar images (EPI) were acquired (TR = 2000 ms, TE = 30 ms, FA = 90°, 64×64×32 matrix at 3.59×3.59×3.85 mm resolution, 33 axial slices). A total of 186 volumes were collected for each run. The first four brain images (T_R_ periods) from each functional run were removed to allow for steady-state tissue magnetisation. High-resolution, T1-weighted structural images were also acquired from each participant (TR = 2200 ms, TE = 4.5 ms, 192×192 matrix×144 slices, at 1.0×1.0×1.25 mm resolution).

### fMRI Analyses

Data were pre-processed and analysed using SPM5 (Wellcome Department of Imaging Neuroscience, Institute of Neurology, London; http://www.fil.ion.ucl.ac.uk/spm), implemented in Matlab (Mathworks Inc., USA). For image realignment, functional images were first realigned to the middle image, applying a six-parameter rigid body transformation to remove the effects of head motion [26]. For spatial normalisation, a mean EPI volume was obtained during realignment, and then aligned to the standard EPI template of SPM5 using nonlinear basis functions. The same registration parameters were then applied to all EPI volumes to register all participants' images to MNI image space. All functional images were spatially smoothed with an isotropic Gaussian kernel of 5 mm, full-width at half-maximum (FWHM).

For first-level analysis, a general linear model (GLM) was used to determine parameter estimates of activation for each condition separately. Event-related regressors were formed modeling activation from the onset of presentation of colour-cue trials (separately for similar and dissimilar responses) and no-go trials, with the standard trials serving as an implicit baseline. To identify brain regions showing greater BOLD signal during performance of dissimilar compared with similar actions we examined the contrast [dissimilar – similar] within every fMRI run. Random-effects group analyses using these contrasts were conducted using a 2×2 factorial model to identify as regions of interest (ROIs) areas showing a significant interaction between the two contexts (Imitative/Complementary) and stimuli (Actions/Arrows). We used a cluster-level threshold of p<0.05, corrected for multiple comparisons, with clusters defined by the voxel-level uncorrected threshold of p<0.001.

To determine the nature of any interaction for the [dissimilar-similar] contrast, we performed post-hoc region of interest (ROI) analyses on % signal change values in brain regions showing an interaction between context (Imitative/Complementary) and stimulus (Actions/Arrows). Values of % signal change were extracted for each experimental condition relative to baseline (no-go trials), averaged across all voxels within ROIs, using the MarsBar toolbox for SPM [27] (available at http://marsbar.sourceforge.net). We plotted % signal change in the ROIs for each of the experimental conditions, and conducted pair-wise comparisons (dependent t-tests) to specifically determine the difference between dissimilar versus similar actions, separately for Action and Arrow conditions in Imitative and Complementary contexts (a total of eight planned comparisons). Note that these pairwise comparisons are independent of the interaction effect that was used to select the ROIs, which rules out the possibility of data distortion due to selection biases [28].
